# Molecular Analysis of Hot-Spot Regions of ACE2 and TMPRSS2 in SARS-CoV-2 “Invulnerable” Individuals

**DOI:** 10.7759/cureus.43344

**Published:** 2023-08-11

**Authors:** Achilleas P Galanopoulos, Zacharoula Bogogiannidou, Styliani Sarrou, Ioanna Voulgaridi, Varvara A Mouchtouri, Christos Hadjichristodoulou, Matthaios Speletas

**Affiliations:** 1 Department of Immunology & Histocompatibility, Faculty of Medicine, University of Thessaly, Larissa, GRC; 2 Laboratory of Hygiene and Epidemiology, Faculty of Medicine, University of Thessaly, Larissa, GRC

**Keywords:** invulnerable individuals, tmprss2, ace2, covid-19, sars-cov-2

## Abstract

Background

Coronavirus disease 2019 (COVID-19) is characterized by a wide clinical variability, ranging from acute illness that may require hospitalization and intensive care unit management to mild and even asymptomatic disease. A more exciting phenomenon is the presence of individuals who came into close contact with COVID-19 patients without prophylaxis but were never infected by SARS-CoV-2, even as an asymptomatic disease.

Aims

We describe four such “invulnerable” individuals and explore if they carry genetic defects in hot-spot regions of *ACE2* and *TMPRSS2* genes, which are responsible for virus entry into the host cells.

Materials and methods

Anti-S humoral and cellular immune responses were evaluated in the study participants through chemiluminescent microparticle immunoassay (CMIA) and enzyme-linked immunosorbent assay (ELISA) and interferon (IFN-γ) secretion measurement, respectively. Moreover, the hot-spot locations of *ACE2* and *TMPRSS2* were analyzed by polymerase chain reaction (PCR) sequencing in order to investigate potential genetic defects.

Results

No pathogenic genetic defects in *ACE2* and *TMPRSS2* were identified in the study participants. However, a functional polymorphism (rs12329760) located in exon 6 of the *TMPRSS2* gene was detected in two of the four participants. In addition, it is worth noting that two individuals displayed adequate humoral and cellular immune responses after COVID-19 vaccination several months after their initial exposure to SARS-CoV-2.

Conclusions

We suggest that *ACE2* and *TMPRSS2* genes are not responsible for the “invulnerable” phenotype against COVID-19.

## Introduction

Since the initial epidemic outbreak in late December 2019 in Wuhan, China, severe acute respiratory syndrome coronavirus 2 (SARS-CoV-2) has spread worldwide. To date, more than 674 million confirmed COVID-19 cases and 6.8 million deaths have been reported, according to the Johns Hopkins Coronavirus Resource Centre (update on February 2023) [1]. SARS-CoV-2 is an RNA betacoronavirus with phylogenetic similarities to SARS-CoV and Middle East respiratory syndrome coronavirus (MERS-CoV), which fall into the same genus [2]. Coronaviruses’ positive-stranded RNA genome encodes for spike (S), nucleocapsid (N), membrane, and envelope proteins [3]. The S protein consists of S1 and S2 subunits, mediating the virus entry into host cells; the S1 subunit can recognize and bind to the host’s receptors, while S2 allows for the fusion between cellular and viral membranes [3]. In particular, the virus infects human cells by binding to the cell surface protein angiotensin-converting enzyme 2 (ACE2) through the receptor-binding domain (RBD) of S1 protein, and then the cellular transmembrane serine protease 2 (TMPRSS2) is required for the priming of S protein and the entry of the virus into host cells [3,4]. The latter is also dependent on the endosomal/lysosomal cysteine proteases cathepsin B and L (CTSB, CTSL), although their activity is likely not essential [5,6].

ACE2 is a carboxypeptidase protein and negative controller of the renin angiotensin system (RAS) and is a critical regulator of blood volume and systemic vascular resistance [7]. It appears to be highly expressed in the small intestine, testis, kidneys, heart, thyroid, and adipose tissue, while medium expression levels are observed in the lungs where upregulation or downregulation events take place in correlation with specific immune signatures [8]. There are two relative protein forms participating in cell pathways [9,10]. The sACE2 form is soluble and can be found in the circulation [9]. The full-length form, mACE2 protein, is located on the cell surface and contains a single extracellular N-terminal domain, a C-terminal membrane anchor, and a conserved zinc-binding domain [10]. As mentioned above, ACE2 is the receptor for S protein of SARS-CoV-2, and the receptor-S1 subunit interaction is crucial for the virus’ entry into the host [10]. The *ACE2* gene is located on the X chromosome and consists of 18 exons [11]. Several genetic positions have been reported for their crucial role in the virus life cycle. It is known that lysine 31 of ACE2 protein (exon 1) is involved in the binding event, interacting with the RBD of S protein [12]. In addition, the arginine and lysine amino acid residues located from 697 to 716 positions of ACE2 are important for its interaction with TMPRSS2 and contribute to efficient enzyme cleavage [13].

TMPRSS2 is a cell surface serine protease anchored in the plasma membrane. Its proteolytic capacities are associated with viral infection, as they activate spike glycoproteins of human coronaviruses (HCoV-229E, HCoV-EMC), fusion glycoproteins of Sendai virus, human metapneumovirus, human parainfluenza viruses (1, 2, 3, 4a, 4b), and hemagglutinin protein of influenza A virus [6,14-17]. It is highly expressed in the prostate, bladder, kidneys, and tissues of the gastrointestinal and respiratory tract [18]. In the lungs, TMPRSS2 expression may be a determinant of viral tropism and pathogenicity of SARS-CoV-2 infection [18]. The *TMPRSS2* gene is expressed in ACE2-positive lung cells [19] and appears to play a remarkable role in coronavirus proliferation in the human respiratory tract [20]. TMPRSS2 affects SARS-CoV-2 infection via two mechanisms; the first is proteolytic cleavage of ACE2, which promotes viral uptake, and the second is cleavage of S protein, which affects the S-ACE2 binding and the virus entry into host cells [13,21]. The *TMPRSS2* gene is located on chromosome 21 and consists of 14 exons [22]. Exon 6 contains a missense variant named rs12329760 (c.478G>A), resulting in the substitution of a valine at site 160 by a methionine (Val160Met), characterized as deleterious in a previous study using bioinformatic techniques [3]. The missense variant contributes to the form of a larger pocket protein, which probably affects the enzyme’s structure and thereby the entry of SARS-CoV-2 into the host cells, as suggested in another computational analysis [23].

SARS-CoV-2 infection (coronavirus disease 2019 [COVID-19]) is characterized by wide variability [24,25]. Thus, some individuals suffer from acute illness requiring hospitalization and intensive care unit management, while the great majority exhibit asymptomatic disease or typical flu-like symptoms [24]. Asymptomatic viral infection is an “uncharted territory” for infectious diseases [25]. Therefore, COVID-19 asymptomatic individuals are invisible and contribute to silent transmission of the disease. The factors contributing to this clinical manifestation are still unknown. In this context, some researchers have suggested that certain individuals may have been exposed to SARS-CoV-2 without leaving a footprint of adaptive immunity because of the efficiency of innate immune mechanisms, or because T cell mediated immunity may affect disease severity and could potentially interpret such cases [25].

An exciting phenomenon is the presence of persons who came into close contact with patients infected by SARS-CoV-2, but they never developed even asymptomatic disease. We could consider these individuals as “invulnerable” since they cannot be infected by the virus. Clearly, the cause of this invulnerability is still obscure.

Among thousands of COVID-19 patients encountered in our area, we identified four individuals who came into contact with the virus, living among COVID-19 patients with no prophylaxis, who were never infected. Considering the important role of ACE2 and TMPRSS2 proteins in the virus life cycle, we hypothesized that genetic defects in the ACE2 binding domain to S protein of SARS-CoV-2, or TMPRSS2 active site crucial for S protein cleavage that drives virus entry into the host cells, may be responsible for disease invulnerability. Therefore, the aim of our study was to explore this possibility in our “invulnerable” individuals.

## Materials and methods

Subjects

Four individuals were analyzed in our study (male/female: 2/2, median age: 43 years). An overview of their medical history and comorbidities are presented in Table 1. All individuals shared the same household and had close contact with COVID-19 patients; three participants (no. 1, 2, and 3 in Table 1) lived in a Roma camp and accompanied their children who were infected by COVID-19 to a non-hospital isolation facility for at least 14 days. The last participant (no. 4 in Table 1) did not display COVID-19 on two occasions, first when her children were infected by SARS-CoV-2 and then when her husband was infected, despite living with her children and husband and being responsible for their attendance without any precaution. All participants displayed repeated negative SARS-CoV-2 molecular tests via reverse transcription polymerase chain reaction (RT-PCR) on nasopharyngeal swabs. All participants provided a signed informed consent; the study was conducted based on the principles of the Helsinki Declaration and was approved by the Medical Faculty of the University of Thessaly (No. 2116).

**Table 1:** Demographic, clinical, and laboratory data of the participants of the study. CMIA, chemiluminescent immunoassay; IFN-g, interferon gamma; F, female; M, male; SARS-CoV-2, severe acute respiratory syndrome coronavirus 2 *The cut-off of positivity was 50 U/mL. ^The cut-off of positivity was 100 mIU/mL

No	Sex	Age (years)	Molecular and/or protein detection of SARS-CoV-2	Anti-N and anti-S anti-SARS-CoV-2 assays	Vaccination	Anti-S IgG after vaccination (CMIA, U/mL)*	Cell-mediated immunity (IFN-g mIU/mL)^
1	M	45	Negative (>2 times)	Negative	Ad26.COV2.S	5915.00	5794.67
2	F	44	Negative (>2 times)	Negative	BNT162b2	443.20	938.56
3	M	39	Negative (>2 times)	Negative	No		
4	F	42	Negative (>4 times)	Negative (twice)	No		

Humoral and cell-mediated responses against SARS-CoV-2

To exclude the possibility that study participants had been infected by SARS-CoV-2 and displayed potential asymptomatic disease, we repeatedly evaluated anti-S and anti-N anti-SARS-CoV-2 antibody responses by enzyme-linked immunosorbent assay (ELISA) and chemiluminescent microparticle immunoassay (CMIA), as described in our previous studies [26,27]. Moreover, the presence of anti-SARS-CoV-2-reactive T cells was estimated according to the secretion of interferon-gamma (IFN-g) in an ELISA assay, after incubation of peripheral blood with S peptides for 20 hours, as described [27]. Using the aforementioned assays, the anti-S humoral and cellular immune responses were also evaluated in two participants who were vaccinated against SARS-CoV-2 with Ad26.COV2.S (Janssen Pharmaceuticals, Beerse, Belgium) and BNT162b2 (Biotech, Mainz, Germany) vaccines, one and a half and four months after vaccination, respectively. Moreover, the presence of total neutralizing antibodies to SARS-CoV-2 was detected after the initial blood sampling using a commercially available kit (cPass SARS-CoV-2 Neutralization Antibody De-tection Kit, USA Inc./Nanjing GenScript Diagnostics Technology Co., Ltd., Nanjing, China), according to the instructions of manufacturer.

Molecular assays

The hot-spot locations of ACE2 and TMPRSS2 were analyzed by PCR sequencing. In particular, genomic DNA was extracted using the Extractme Genomic DNA Kit (Blirt, Gdansk, Poland), according to manufacturer’s instructions. Three exons of ACE2 (exon 1 including the amino acid residue lysine at position 31; exons 16 and 17 including the arginine and lysine amino acid residues 697 to 716, and four exons of TMPRSS2 (exon 6 including the common functional polymorphism rs12329760; exon 9 including the histidine residue 296; exon 10 including the aspartic acid residue 345; exon 13 including the serine residue 441) were amplified by PCR, as presented in detail in Table 2.

For all PCR reactions, a total of 100 to 200 ng of DNA was amplified in a 30-mL reaction mixture using 62.5 mM each deoxynucleotide triphosphate, 500 pmol of each primer, 1.5 mM of MgCl2, and 0.8 U of DFS-Taq DNA polymerase (Bioron GmbH, Romerberg, Germany) in a buffer supplied by the manufacturer. Afterward, the PCR products were purified using a PCR purification kit (Macherey-Nagel, Duren, Germany) and/or extraction system (Macherey-Nagel) and were directly sequenced using an ABI Prism 310 genetic analyzer (Applied Biosystems, Foster City, CA).

**Table 2:** PCR protocol information for targeted exons of ACE2 and TMPRSS2 genes.

Gene	Primers	Sequence	PCR conditions	PCR product
ACE2				
Exon 1	Forward	5’-GGCCATAAAGTGACAGGAGAGGTAAGG-3’	94°C for 2 min followed by 30 cycles (94°C for 30 s, 60°C for 30 s, and 72°C for 30 s) and a final elongation at 72°C for 5 min	600 bp
	Reverse	5’-TGGAGGCAAACATCCAATCTCA-3’		
Exon 16	Forward	5’-TCTTCATTCTCTTGATTGTGTCCTCTG-3’	94°C for 2 min followed by 30 cycles (94°C for 30 s, 60°C for 30 s, and 72°C for 30 s) and a final elongation at 72°C for 5 min	230 bp
	Reverse	5’-TTGTTGTATTTGGTAACCCCCTCAC-3’		
Exon 17	Forward	5’-TCTAAGTGTCCCCTTTGCTGTTTT-3’	94°C for 2 min followed by 30 cycles (94°C for 30 s, 60°C for 30 s, and 72°C for 30 s) and a final elongation at 72°C for 5 min	252 bp
	Reverse	5’-TAGGAAAGGCCACTTACTTCTTCC-3’		
TMPRSS2				
Exon 6	Forward	5’-AAGAAACTCATGGATAATCCTCCCTCT-3’	94°C for 2 min followed by 30 cycles (94°C for 30 s, 60°C for 30 s, and 72°C for 30 s) and a final elongation at 72°C for 5 min	274 bp
	Reverse	5’-ACAATTGTCCCCAGCACTCAT-3’		
Exon 9	Forward	5’-GCATCCTCCCTTCCCCAAGAG-3’	94°C for 2 min followed by 30 cycles (94°C for 30 s, 62°C for 30 s, and 72°C for 30 s) and a final elongation at 72°C for 5 min	534 bp
	Reverse	5’-AGTGCATACAGCCATGCACTGC-3’		
Exon 10	Forward	5’-GCTCTGTGTTTATGGCTCTGAGAT-3’	94°C for 2 min followed by 30 cycles (94°C for 30 s, 62°C for 30 s, and 72°C for 30 s) and a final elongation at 72°C for 5 min	274 bp
	Reverse	5’- CATCTCTTTAAGATTCTGCCAACCTG-3’		
Exon 13	Forward	5’-TGCGGCAGATTGTGACTTGA-3’	94°C for 2 min followed by 30 cycles (94°C for 30 s, 62°C for 30 s, and 72°C for 30 s) and a final elongation at 72°C for 5 min	272 bp
	Reverse	5’-AACAGATGTCTGGCTTTGGCT-3’		

## Results

As presented in Table 1, the repeated serological assays, including neutralizing anti-SARS-CoV-2 antibodies, were negative in all study participants. Furthermore, anti-S cellular immune responses were also evaluated in a participant (No. 4 of Table 1) following her second exposure to SARS-CoV-2, along the aforementioned humoral responses; however, they were also negative (anti-S IgG levels: 5.4 U/mL, cut-off of positivity: 50 U/mL; IFN-g levels: 65.38 mIU/mL, cut-off of positivity: 100 mIU/mL). Interestingly, individuals vaccinated against SARS-CoV-2 (No. 1 and 2 of Table 1) displayed adequate anti-S humoral and cellular responses, as presented in detail in Table 1.

**Figure 1:**
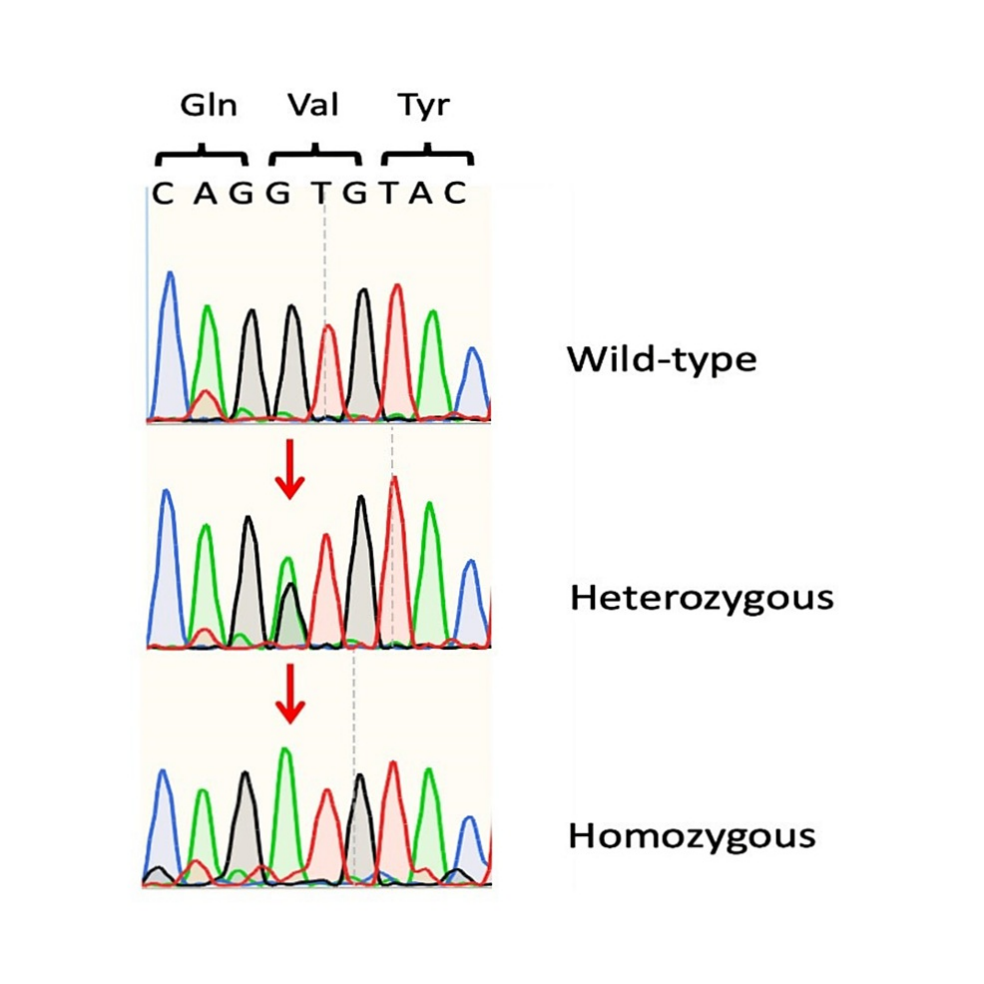
Demonstration of the polymorphism rs12329760 (c.478G>A, p.Val160Met) in our study participants.

No pathogenic genetic defects in ACE2 and TMPRSS2 were identified in the study participants (Table 3). However, two participants carried the functional polymorphism rs12329760 in homozygous and heterozygous state, respectively (Table 3, Figure 1). Additional common synonymous and intronic variants were found and presented in detail in Table 3.

**Table 3:** Molecular results of the study participants. Heter, heterozygous; Homo, homozygous; wt, wild-type rs12329760: c.478G>A, p.Val160Met, allele frequency 0.244 (ExaC) [https://www.ncbi.nlm.nih.gov/snp/rs12329760] rs2298659: c.777C>T, p.Gly259=, allele frequency 0.259 (ExaC) [https://www.ncbi.nlm.nih.gov/snp/rs2298659] rs17854725: c.768T>C p.Ile256=, allele frequency 0.560 (ExaC) [https://www.ncbi.nlm.nih.gov/snp/rs17854725] rs73230068: c.899+85C>G, allele frequency 0.035 (gnomAD-Genomes) [https://www.ncbi.nlm.nih.gov/snp/rs73230068]

No	ACE2			TMPRSS2			
	Exon 1	Exon 16	Exon 17	Exon 6	Exon 9	Exon 10	Exon 13
1	wt	wt	wt	wt	rs2298659-Heter rs73230068-Heter	wt	wt
2	wt	wt	wt	rs12329760-Homo	rs2298659-Heter	wt	wt
3	wt	wt	wt	rs12329760-Heter	rs2298659-Heter rs17854725-Heter	wt	wt
4	wt	wt	wt	wt	rs17854725-Homo	wt	wt

## Discussion

It has been widely observed that some individuals have been exposed for an extended period and without applying personal protective measures to persons infected by SARS-CoV-2, however, they never develop symptomatic COVID-19. In fact, the majority of these individuals have been infected by the virus and display an asymptomatic course of the disease without signs or symptoms of COVID-19, but they can still transmit the virus to others. Such cases have been confirmed by the emergence of anti-SARS-CoV antibodies in their serum over time [25]. However, anecdotal cases exist, which are similar to our study participants, who were never infected by the virus and whose immunological tests do not indicate a past infection.

Such “invulnerable” cases were included in our study. These individuals never displayed a positive molecular and/or antigenic test for SARS-CoV-2 and never developed antibodies against SARS-CoV-2 despite the fact that they were exposed to the virus for a long time period without protective measures. However, two of these individuals developed adequate immune responses after vaccination. Thus, we hypothesized that these individuals may be protected from the virus due to its “inability” to enter into their host cells. Consequently, we analyzed the hot-spot regions of *ACE2* and *TMPRSS2 *genes that are crucial for this process, but no pathogenic genetic defects were found.

Interestingly, we observed that two participants of our study carried the functional polymorphism rs12329760 (c.478G>A, p.Val160Met), which is located into exon 6 of the TMPRSS2 gene (Table 3, Figure 1). As mentioned above, this missense variant is predicted to be damaging by PolyPhen-2 and deleterious by SIFT bioinformatic techniques, since it is located at the exonic splicing enhancer srp40 site and results in weakened protein stability [3]. Previous studies have suggested that this missense variant is significantly associated with a genetic susceptibility to COVID-19 and a less severe disease, especially in homozygotes [3,28]. In our study, this polymorphism was not present in all participants, suggesting that it is not the key culprit of their “invulnerable” phenotype.

Therefore, the reason for the “invulnerable” phenotype of our individuals is still obscure. A possible explanation may be the cross-reactivity phenomenon, namely the presence of neutralizing antibodies and/or T cells from previous infections against other pathogens that can react against SARS-CoV-2. Indeed, cross-reactive antibodies and T cells are known to play crucial protective roles in host responses to viral infections. Such a phenomenon has already been described in a patient infected by SARS-CoV in the past, displaying neutralizing antibodies against SARS-CoV-2 as well [29]. In addition, 20% to 50% of unexposed individuals around the world seem to have acquired T cell clones recognizing SARS-CoV-2 antigens, due to past infections by other human coronaviruses; this, in fact, may affect their susceptibility to COVID-19 disease [30]. It is possible that such neutralizing antibodies and/or T cell clones may be protective against SARS-CoV-2 in our participants and remains to be explored in further studies.

A limitation related to this study concerns the sample size, since the number of our “invulnerable” individuals was rather small. However, the discovery and the further study of such cases is rare and difficult, as several individuals initially thought to be “invulnerable” were actually infected in an asymptomatic manner, as demonstrated by the detection of anti-N antibodies in their serum after their contact with SARS-CoV-2. Additionally, although we targeted four exons of TMPRSS2 and three exons of ACE2, as they are crucial locations for viral endocytosis, a large-scale genetic analysis (i.e. a whole exome sequencing approach) could potentially strengthen our findings and will further elucidate the genetic background of these “invulnerable” individuals.

## Conclusions

In this study, we describe four individuals who came into close contact with SARS-CoV-2, living with COVID-19 patients with no prophylaxis, but were never infected. The genetic analysis of hot-spot regions of *ACE2* and *TMPRSS2 *genes did not reveal any pathogenic genetic defect, suggesting that these genes are not responsible for this “invulnerable” phenotype. Alternative interpretations of this observation must be considered, such as the cross-reactivity phenomenon. Further investigation in this field should be conducted.
